# Aluminum Conductor Steel-Supported Conductors for the Sustainable Growth of Power Line Capacity: A Review and Discussion

**DOI:** 10.3390/ma17184536

**Published:** 2024-09-15

**Authors:** Milad Jalilian, Jordi-Roger Riba, Pooya Parvizi

**Affiliations:** 1 Department of Physics, Faculty of Science, Lorestan University, Khorramabad P.O. Box 465, Iran; jalilianm70@gmail.com; 2 Pooya Power Knowledge Enterprise, Tehran 1466993771, Iran; 3 Department of Electrical Engineering, Universitat Politècnica de Catalunya, 08222 Terrassa, Spain; 4 Department of Mechanical Engineering, University of Birmingham, Edgbaston, Birmingham B15 2TT, UK; pxp046@student.bham.ac.uk

**Keywords:** aluminum conductor steel-supported, ACSS, high-capacity conductors, high temperature low sag, power transmission lines

## Abstract

Industrial development and population growth have increased the need for higher-capacity power transmission lines. Aluminum conductor steel-supported (ACSS) conductors, a type of high-temperature low-sag (HTLS) conductor, are now widely used in new designs and reconductoring applications. ACSS conductors are preferred over traditional aluminum conductor steel-reinforced (ACSR) conductors due to their high strength, low sag, and excellent thermal stability. These attributes have garnered significant interest from researchers, engineers, and manufacturers. This paper provides a comprehensive review of the structure, properties, testing methods, and environmental behavior of ACSS conductors.

## 1. Introduction

Electricity is crucial for the well-being of citizens and the social prosperity of developed economies. Over the last two decades, the scientific community has shown a growing research interest in various perspectives related to electricity WW [1]. The occurrence of energy losses along power delivery network has persistently captured the attention of researchers. There are various ways to improve transmission line efficiency and reduce losses including uprating [2,3,4,5], reconductoring [6], dynamic line rating [7,8,9,10,11], energy storage integration [12,13], dynamic thermal rating, network topology optimization [14,15], and real-time online analysis [16,17]. Overhead conductors are cost-effective and reliable for transmitting high amounts of electrical power over long distances. They are easy to repair, flexible, and have a longer lifespan compared to underground cables. This makes them a versatile and important component of the power industry. ACSR conductors are the most widely used type of conductor in power transmission lines. They have a central steel core surrounded by several layers of aluminum strands. The steel core provides the conductor with strength and support, while the aluminum strands provide good electrical conductivity. ACSR conductors sag excessively at high temperatures, which limits their ability to meet the growing demand for electricity and transmission capacity. The increasing popularity of HTLS conductors for high-voltage lines presents a favorable choice for replacing ACSR conductors and enhancing the responsiveness of the power supply network [2,18,19,20,21,22,23]. While conventional conductors typically operate at temperatures below 75 °C, ACSS conductors have the capability to operate continuously at temperatures of at least 150 °C [3,24]. The most important types of high-capacity conductors (HTLS) based on their brand are ACSS, super-thermal-resistant aluminum conductor invar-reinforced (ZTACIR), gap type aluminum conductor steel-reinforced (GTACSR), aluminum conductor composite-reinforced (ACCR), and aluminum conductor composite core (ACCC) [3,4,5,6,25,26,27]. ACSS conductors are the right choice to meet the growing needs of modern power transmission systems where higher current-carrying capacity is needed [28]. ACSS conductors were first introduced in 1970. At that time, the name of this conductor was steel-supported aluminum conductor (SSAC) [29,30]. ACSS conductors have garnered significant attention due to their unique design and benefits over conventional conductors. Made of high-strength aluminum alloy strands wrapped around a central steel core, ACSS conductors improve current-carrying capacity, reduce thermal expansion effects, and enhance overall system reliability [3,31,32]. This review highlights the construction, material properties, production processes, and key considerations related to ACSS conductors. Moreover, the study delves into critical factors such as creep behavior, stress–strain characteristics, short-circuit current handling capability, electrical resistance, self-damping, current–temperature relationships, corona discharge, sag–temperature behavior, lightning, galloping, aeolian vibrations, fretting fatigue, environmental impacts, and sustainable development.

## 2. Construction and Materials

ACSS conductors consist of fully annealed aluminum or aluminum-alloy wires stranded around a steel core, which may be galvanized, aluminum-clad, or Galfan-coated. The wires have either a circular or trapezoidal cross-section [33,34,35,36,37]. High electrical conductivity in aluminum wires is crucial, necessitating the use of electrical conductor (EC)-grade aluminum with a minimum purity of 99.5% [38]. Balancing mechanical strength, electrical conductivity, and high-temperature operation in aluminum conductors is challenging due to the inverse relationship between strength and conductivity [39]. Aluminum alloys with suitable properties can address this issue. Studies have explored the use of alloying elements like zirconium, scandium, yttrium, and erbium to achieve an optimal combination of strength, conductivity, and thermal stability [33,39,40,41,42,43,44,45,46]. Elements like Zr and Sc control grain sizes in recrystallization and enhance thermal resistance, increasing strength and allowing operation at temperatures of 150–230 °C [33,44]. However, these alloying elements can reduce electrical conductivity, which can be improved with appropriate heat treatments [41,42]. For example, an Al-0.2Y-0.2Sc alloy shows a tensile strength of 199–202 MPa and an electrical conductivity of 60.8–61.5% IACS, with thermal stability up to 550 °C [47]. Another alloy, Al-0.09Sc-0.12Zr-0.07Si-0.07Fe, has a tensile strength of 210 MPa, an electrical conductivity of 60.2% IACS, and thermal resistance up to 400 °C. The alloying elements can be added in the furnace, followed by hot rolling, solution heat treatment, and aging treatment either before or after cold wire drawing [41]. Steel core wires meet ASTM standards for coatings: “GA” (galvanized), “MA” (Galfan), and “AW” (aluminum-clad). Strength levels are indicated by “GA2” (typical), “GA3” (high strength), “GA4” (extra-high strength), and “GA5” (ultra strength) [48,49,50,51,52]. The chemical compositions for these steel wires in ACSS conductors are detailed in Table 1.

Galvanized coating (GA) is the simplest and most cost-effective option with decent corrosion resistance, but its resistance and maximum allowable temperature are limited. Newer coatings like Galfan (MA) and aluminum cladding offer improvements. Galfan coating enhances corrosion resistance and thermal stability up to 250 °C, preserving mechanical properties and extending ACSS conductor service life. Aluminum-clad steel (AW) core wire adds strength, corrosion resistance, and excellent electrical conductivity, ideal for harsh environments and coastal areas [25,30,53].

## 3. Mechanical, Physical, and Thermal Properties of Aluminum Strands and Steel Cores

As per the guidelines set forth by ASTM and BS EN standards [48,49,50,51,52,54,55,56,57,58,59], the reinforcing steel wires utilized within the core of overhead conductors are mandated to satisfy distinct physical, mechanical, and electrical criteria. These encompass parameters such as density, electrical conductivity, coefficient of linear thermal expansion, tensile strength, tensile stress at 1% elongation, and overall elongation. A consolidated summary of these specifications is presented in Table 2.

## 4. ACSS Conductor Production Process

As mentioned earlier, ACSS conductors are composed of aluminum strands wound around a steel core made up of coated steel wires. These aluminum and steel wires are produced separately through distinct processes. Once both the aluminum wires and steel core are prepared, the aluminum strands are stranded around the steel core, resulting in the final conductor.

### 4.1. Aluminum Wires Production Process

The aluminum wire production process involves the Properzi casting process, which includes melting in the main furnace, transfer to the holding furnace, crystal production, hot rolling, and aluminum rod production [60,61]. These rods are then drawn through dies using lubricants to form circular or trapezoidal wires. To improve their electrical properties, the wires undergo annealing during drawing. One of the challenges in the Properzi process is to minimize waste and ensure that the quality of the product remains high. The casting process is complex and requires accurate control of all parameters as well as a deep understanding of the process to produce a high-quality product. The main casting defect to be encountered is the shrinkage cavity defect, which scrapes many coils to avoid quality problems during the drawing process, which later translates into loss of sales, but it has been shown that improving the quality of the cast bar could lead to reduced scrap and produce superior aluminum rods [62]. Manufacturers of electrical-grade aluminum also employ diverse methods to enhance purity and conductivity [63]. Transition metal impurities such as V, Ti, Zr, and Cr lead to a decrease in the electrical conductivity of smelter-grade aluminum. A commonly used technique entails adding boron into the molten aluminum. The boron triggers a reaction with vanadium and titanium, forming borides. These transition metals can reduce the electrical conductivity of aluminum. As a result of this reaction, TiB_2_ and VB_2_ compounds are generated, which subsequently gather and settle in the holding furnace [64,65,66,67,68]. The standard chemical composition of aluminum for electrical purposes is given in Table 3.

### 4.2. Steel Wire Production Process

#### 4.2.1. Aluminum-Clad Steel Wires

**Figure 1:**
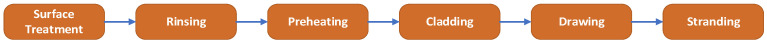
The production process of aluminum-clad steel wires (AW).

To manufacture aluminum-clad steel wires, a cladding process is used, starting with surface cleaning via mechanical, chemical, and ultrasonic treatments to ensure proper bonding. After water rinsing, the wires are preheated and coated with semi-solid aluminum, resulting in aluminum cladding. The cladded wire is then drawn to the desired dimensions using dies and dry-powder lubricants, and the aluminum-clad core is produced using a stranding machine The production process is visually represented in Figure 1. Uniformity and surface quality issues are common challenges in the production of aluminum-clad steel wire. However, a recently proposed production method that synergistically combines solid–liquid composite formation has been shown to improve uniformity, surface quality, mechanical properties, and energy efficiency [69]. Key quality parameters are surface treatment, line speed, and preheating temperature. Aluminum-clad steel cores have similar mechanical properties to galvanized steel cores but are more resistant to high temperatures, maintaining integrity up to 300 °C before tensile strength decreases [70].

#### 4.2.2. Galvanized Steel Wires

**Figure 2:**
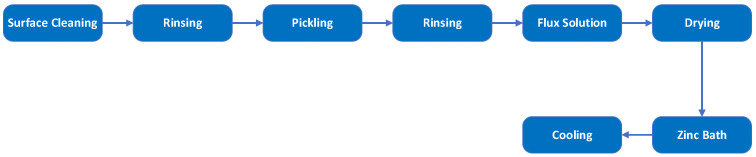
The production process of galvanized steel wires (GA).

Galvanized wires are the predominant choice for the core of conventional overhead conductors, especially ACSR conductors. The galvanization process is a conventional method used to protect ferrous metals against corrosion. This process involves several sequential stages: cleaning, rinsing, pickling, rinsing again, applying a flux solution, drying, immersing in a zinc bath, and subsequent cooling. The zinc bath temperature can reach up to 450 °C. Proper surface preparation is crucial, as inadequate cleaning can result in an uneven, discontinuous surface texture unsuitable for use in overhead conductors [71,72]. The continuous hot-dip galvanizing process is shown in Figure 2. Key parameters for quality galvanizing include surface pretreatment, dipping techniques, and post-treatment [73]. Cracking in the zinc coating, peeling, and poor adhesion are primary defects in continuous hot-dip galvanizing. Recent findings indicate that these issues can be minimized by optimizing the zinc bath temperature, adjusting the immersion time, and carefully controlling the cooling rates [74]. Alloying elements such as C, Si, P, and Mn can affect the galvanized coating by influencing the growth of the Fe-Zn phase [75]. Elevated carbon content accelerates the dissolution rate of iron into the melt, leading to a fragile and unstable ζ phase coating [72,76]. To enhance bonding between the zinc and the steel substrate, a small amount of aluminum (approximately 0.05 wt. % to 0.25 wt. %) may be added to the mixture [77]. The zinc bath temperature significantly affects the coating thickness, varying between 450 °C and 530 °C. Verma et al. [78] found the highest coating thickness at 530 °C, while Biaco et al. reported maximum thickness at 480 °C. However, the ζ phase formed at these temperatures is often incoherent and unstable, making it unsuitable for industrial use [75]. The zinc coating on steel core wires limits high-temperature operation. Above 200 °C, the coating’s adhesion weakens, reducing corrosion resistance and lifespan due to pitting. Over 225 °C, zinc can alloy with steel, forming brittle compounds that flake off, further reducing corrosion resistance and increasing fatigue susceptibility [70].

#### 4.2.3. Galfan-Coated Steel Wires

**Figure 3:**
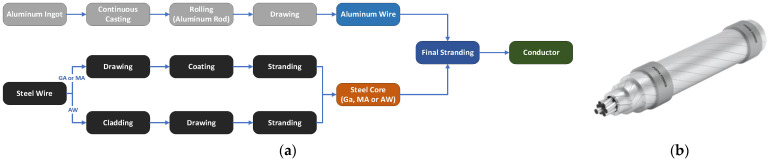
(**a**) Production diagram of an ACSS conductor and (**b**) schematic of an ACSS conductor.

Galfan-coated steel wires are used in the core of overhead conductors. The production process of Galfan-coated steel wires is similar to that of galvanized steel wires, with the primary difference being their chemical compositions. While galvanized coatings are predominantly zinc, Galfan coatings contain about 5% aluminum, enhancing their anti-corrosive properties. Additionally, a small amount of mischmetal, including rare-earth elements like cerium and lanthanum, is added to improve flowability and wettability. Galfan coatings offer significant advantages such as ductility, impressive corrosion resistance, and a reduction in bare spots. These attributes make Galfan-coated wires an excellent choice for applications requiring robust corrosion resistance [79]. Unlike the typical hot-dipped galvanizing process, this unique coating is specifically applied to the core wires to endure extended operational temperatures of up to 300 °C, allowing conductor temperatures around 250 °C [70]. Galfan-coated wires are advantageous in scenarios necessitating enhanced corrosion resistance and long-term durability at high operational temperatures. Key factors in the Galfan coating process include surface pretreatment, flux type, Galfan bath composition, and post-treatment. After coating, aluminum wires are stranded around the steel core to complete the conductor, followed by a final inspection. Figure 3 provides a visual representation of the production process.

## 5. Performance Characteristics and Environmental Behavior of ACSS Conductors

### 5.1. Current-Carrying Capacity and Transmission Losses

ACSS conductors are engineered to increase the maximum current-carrying capacity of transmission lines by enabling operation at elevated temperatures, reaching up to 250 °C, while maintaining their structural strength [80]. The steel core of ACSS conductors supports the mechanical load, allowing the aluminum strands to operate effectively at high temperatures without a significant reduction in tensile strength. This design mitigates the sag commonly observed during high-temperature operation, thereby maintaining required clearances and improving overall system reliability. In addition, trapezoidal wires can be used in ACSS conductors, which reduces the diameter of the conductor, resulting in lower wind and ice loads. This, in turn, increases the current-carrying capacity and reduces transmission losses. ACSS conductors have excellent thermal stability, allowing them to maintain optimal conductivity even under high-temperature conditions. This characteristic enables ACSS conductors to effectively handle increased power loads, resulting in reduced resistive losses and improved power transmission efficiency [53].

### 5.2. Creep Behavior

Conductor creep refers to the permanent stretching of a conductor caused by pulling forces, leading to increased sag. Safety margins are necessary to ensure adequate ground clearance throughout the conductor’s lifespan. Creep can be categorized into two types: geometrical settlement and metallurgical deformation [81]. Geometrical settlement occurs due to high conductor tensions during severe weather conditions. When strands draw closer, plastic deformation happens at the points where strands from different layers intersect or contact each other [82]. This phenomenon primarily affects soft metals like aluminum and impacts the entire conductor. Although this type of creep is not time-dependent, it occurs within the first hour of high tension. Prolonged high tension leads to metallurgical deformation instead [81,83]. Metallurgical deformation results from mechanical and thermal stress over time [82,84]. There are two forms: normal-temperature (long-term) creep and elevated-temperature creep. Elevated-temperature creep occurs rapidly during emergency high-temperature conditions, impacting untreated aluminum alloys used in conductors. Pre-tensioning effectively eliminates long-term creep in ACSS conductors [70,81]. Traditional conductors undergo long-term creep, whereas ACSS conductors do not exhibit creep during their service life. This is because all stress within the aluminum quickly transfers to the steel core during operation. Consequently, aluminum creep does not affect the ultimate sag of ACSS conductors. The steel core has an exceedingly minimal long-term creep rate, negligible under normal stress conditions [53]; therefore, creep is not a factor for ACSS conductors [85]. The phenomenon of creep in overhead conductors leads to several critical issues, such as an increase in sag, which can compromise safety clearances, and a decrease in tension, which affects mechanical stability. Continuous creep also reduces the strength of the conductor, making it more susceptible to external stresses such as wind or ice. Conductor creep can be mitigated by two methods, pre-tensioning [83] and over-tensioning [86]. Pre-tensioning refers to the temporary application of tension to the conductor prior to installation, which helps prevent early creep. Studies show that since ACSS conductors have annealed aluminum wires, pre-tensioning can be very beneficial and removes most of the geometrical settlement before the final clamping. This is because, after aluminum creep occurs, the tensile stresses in the conductor are transferred to the core [81]. On the other hand, over-tensioning involves installing the conductor at a higher tension level to compensate for future creep. These approaches are often used together to maximize efficacy, especially for HTLS conductors. These conductors must be carefully managed to ensure that stress is evenly distributed among their components, resulting in long-term stability and performance. Despite ACSS conductors operating at high temperatures, there are no specific standard test methods for evaluating creep at high temperatures for them. Developing a dedicated testing standard is necessary to understand the creep behavior of ACSS conductors better.

### 5.3. Stress–Strain

A stress–strain test can be conducted on both the complete conductor and the steel core individually to evaluate the tensile characteristics of the conductor. This test adheres to the guidelines outlined in the BS EN 50540 standard [55]. The procedure involves applying varying loads and employing different load-holding durations. The results from tensile stress–strain tests provide a visual representation of how different conductors, commonly employed in bare overhead transmission lines, actually perform under tension. These tests also reveal how certain factors, such as repeated stressing and the cable’s lay (the arrangement of individual wires in the cable), influence the behavior of these conductors [87]. By repeating this process, a stress–strain curve is generated, allowing for the determination of the conductor’s tensile behavior, as well as the initial and ultimate moduli [55].

### 5.4. Short-Circuit Current

A short circuit can result from excessive current flow, accidental incidents, equipment failures, insulation breakdowns, or adverse weather conditions. This event causes a massive surge in electric current, leading to power outages, damaged circuit devices, fire hazards, and explosions. The excessive heat generated can cause overcurrent flow, rapidly increasing the conductor’s temperature. In aluminum wires, this can lead to a phenomenon called “bird caging”, where wires become distorted and twisted. Proper safety measures and preventive maintenance are crucial to mitigate these risks [88]. During a short-circuit test, conductor temperature difference (initial and final) can be of approximately 60 °C [89]. The short-circuit test is a crucial assessment conducted on an HTLS conductor that is subjected to a short-circuit current for a specified duration [90,91]. The test measures the conductor’s thermal performance, including its resistance to overheating and potential damage caused by the generated heat. This information is vital for determining the conductor’s suitability for real-world applications, ensuring its reliability and safety in power transmission systems [92]. As there are no specific standard test methods for the short-circuit test of ACSS conductors, the short-circuit test method used for ACSS conductors is IEC 60794, which is designed for optical ground wire (OPGW) conductors. A dedicated short-circuit test method for ACSS conductors is needed.

### 5.5. Electrical Resistance

#### 5.5.1. DC Electrical Resistance

The direct current (DC) resistance of the entire conductor is determined by several factors, including the quantity of aluminum and steel wires, the cross-sectional area of each wire, the respective electrical resistivity of aluminum and steel, the prevailing temperature, and the increase in resistance due to the stranding process. Since electrical resistance is temperature-dependent, the DC resistance should be specified at 20 °C. The DC resistance per unit length of the conductor can be calculated using Pouillet’s law. The electrical resistance of all aluminum strands of unit length is as follows:

(1) $$R_{Al}=\frac{r_{Al}}{N_{Al}}\times P_{Al}$$

where $N_{Al}$ is the number of aluminum wires used in the conductor, and $P_{Al}$ is the stranding increment of aluminum strands due to stranding given in ASTM and BS EN [55,93,94]. The electrical resistance of all aluminum strands is as follows:

(2) $$R_{st}=\frac{r_{st}}{N_{st}}\times P_{st}$$

where $N_{st}$ is the number of steel wires in the conductor’s core, and $P_{st}$ is the stranding increment of steel wires due to stranding and given in ASTM and BS EN [55,93,94]. As the outer aluminum strands and the inner steel core are parallel, the overall conductor’s electrical resistance of unit length will be as follows:

(3) $$R_{DC}=\frac{R_{Al}\times R_{st}}{R_{Al}+R_{st}}$$

If the electrical resistance at any other temperature ($T_{2}$) than 20 °C is needed, it can be calculated as follows:

(4) $$R_{DC}\left(T_{2}\right)=R_{20\;{}^{\circ}C}\times \left(1+\alpha \left(T_{2}-20\right)\right)$$

where α is the temperature coefficient of resistivity, which depends on the type of material.

#### 5.5.2. AC Electrical Resistance

While the DC electrical resistance is determined in a specific temperature (T), the AC electrical resistance can be obtained in that temperature. First, the X value shall be determined as follows:

(5) $$X=\frac{0.4497}{\sqrt{{R(T)}_{DC}}}$$

Then, based on the obtained X value, the corresponding K values determined according to the K−X table found in [95], and finally using the following equation, the corresponding AC resistance is calculated using the following formula:

(6) $$R{\left(T\right)}_{AC}=K\times R{\left(T\right)}_{DC}$$

### 5.6. Self-Damping Behavior

As conductors flex, internal movement between strands generates functional forces that contribute to damping [81,96]. Metallurgical damping within the core and individual strands further adds to this effect. Combined, these dissipative mechanisms form conductor self-damping, crucial for absorbing energy during aeolian vibrations [97]. Increased conductor tension reduces strand slippage, lowering self-damping and potentially intensifying aeolian vibrations, thereby raising fatigue risks. Maintaining lower tensions helps mitigate this effect. Frequency and magnitude of vibration also impact self-damping [98], with research showing larger conductors dissipate more power. Trapezoidal conductors tend to dissipate more power at moderately high frequencies (>20 Hz) compared to similarly sized ACSR conductors [99]. As the load is totally transferred to the core after reaching the knee point temperature, the self-damping of ACSS conductors improves at higher operating temperatures [81]. The absence of studies concerning self-damping of ACSS conductors poses a challenge within this field.

### 5.7. Current–Temperature Calculation

The temperature and current-carrying capacity of conductors are correlated and influenced by environmental factors such as wind speed, direction, ambient temperature, precipitation, and solar radiation. Transmission line power transfer capacity is limited by stability, voltage, and thermal constraints, with thermal limits being crucial for safety and reliability. Elevated conductor temperatures reduce their current-carrying ability, making it important to forecast and simulate this correlation. Understanding conductor heating and cooling processes is vital, as higher wind speeds increase transmission capacity by cooling distribution lines [100,101,102]. Techniques used to calculate the current–temperature relationship are essential tools for determining how much electrical current overhead conductors can safely carry at their designated operating temperature. These methods also serve as a basis for estimating factors like sag performance, creep, annealing rate, and tensile strength reduction. Inaccuracies in these calculations can impact these factors. Several physical phenomena affect current–temperature calculations in overhead conductors, including Joule heating, solar heating, corona heating, and cooling mechanisms like convective, radiative, and evaporative cooling [103,104]. Joule heating occurs when an electric current generates thermal energy within a conductor. Solar heating results from the conductor absorbing heat from the sun’s magnetic wavelengths. Corona heating adds thermal energy due to partial discharge in the ionized air near the conductor’s surface. Convective cooling transfers heat from the conductor’s surface via wind, influenced by the Reynolds number. Radiative cooling emits heat from the conductor as electromagnetic waves [103]. Evaporative cooling happens when water on the conductor’s surface evaporates, cooling the conductor [105]. Each of these mechanisms can be modeled using different approaches. From the heat balance, the temperature and the maximum electrical-current-carrying capacity can be determined. The heat balance equation is as follows:

(7) $$\underset{Heating}{\underbrace{P_{J}+P_{S}+P_{i}}}=\underset{Cooling}{\underbrace{P_{C}+P_{R}+P_{w}}}$$

The heating mechanisms of Joule heating, solar heating, and corona heating are denoted as $P_{J}$, $P_{S}$, and $P_{i}$, respectively. On the other hand, the cooling mechanisms of convective cooling, radiative cooling, and evaporative cooling are represented by $P_{C}$, $P_{R}$, and $P_{w}$, respectively. These mechanisms can be modeled by various standards and approaches, such as those by IEEE, IEC, and Cigre [106,107].

### 5.8. Corona Effect

Corona is a non-linear phenomenon occurring during the initial stage of electrical discharges, where electric current passes from a conductor to the ionized surrounding medium [108]. This involves localized discharges near electrode surfaces with intense electric fields, challenging the insulating capabilities of the surrounding dielectric medium. First meticulously examined by Faraday in 1838, the corona phenomenon releases electrical energy when the electric field around a conductor ionizes the neighboring air, creating a halo or visible glow [109]. Corona discharge in transmission lines can produce audible hissing sounds and the distinctive scent of ozone, generated through the breakdown and recombination of O_2_ molecules. The glow’s appearance and arrangement depend on the phase of the AC signal at any specific point in time [110]. Both microscopic and macroscopic approaches can study and simulate corona. High voltage in overhead power lines can induce corona discharge, increasing energy dissipation due to line design and operational factors. Higher voltages can exceed air’s insulation capacity near conductors, causing localized discharge and potentially increasing losses per unit length (p.u.l.) [111]. While often seen as undesirable, the corona effect can be beneficial during lightning-induced surges, reducing peak traveling wave values and slowing their propagation by enhancing line capacitance, thus protecting critical electrical devices and improving system resilience against transient overvoltages [112]. Li et al. [113] studied the effect of water droplet sizes on corona inception and conductor vibration relative to power frequency phases, noting that uniform, stable droplets synchronize vibration and corona emissions over extended lengths. Xin Qi et al. [114] examined surface contaminants like kaolin, NaCl, and carbon, finding that uniform coatings weaken corona discharge by reducing dielectric barrier discharge intensity and surface electric field. Contaminants increase onset voltage and decrease photon counting rate, DC electric field, and ion current density, with electrically conductive contaminants having a smaller impact compared to coating thickness. Jiahui Zhu et al. [115] investigated how conductor surface morphologies under positive DC voltages affect corona behavior, discovering that higher voltage and longer testing create distinctive surface patterns, increasing roughness and enhancing corona discharge. Pei Xu et al. [116] applied a TiO_2_ coating using plasma spraying on conductor surfaces, improving surface quality and reducing roughness (Ra). However, while bare wire surfaces remained stable, coated surfaces degraded due to photocatalysis facilitated by the electric field, despite overall improvement in surface quality. Megala et al. [117] found that wet conductor surfaces have a higher corona inception voltage compared to dry surfaces, using an indoor corona cage. Increasing the conductor diameter helps mitigate corona loss. DC transmission lines exhibit lower corona loss than high-voltage AC lines due to higher material conductivity, which reduces corona effects.

### 5.9. Sag–Temperature Behavior

Once the conductor is under operational stress or pre-stressed during installation, its sag primarily depends on the length changes in the steel core due to temperature fluctuations. The sag of a conductor can be estimated using mathematical models. For a conductor installed between two level spans that are relatively close, the sag can be approximated by the following parabolic equation [118]:

(8) $$D=\frac{wS^{2}}{8H}$$

where D is the sag of the conductor, S is the span length, w is the weight of the conductor per unit length, and H is the horizontal component of the tension force. In non-uniform conductor designs, where the core material differs from the outer layers, the conductor heats up under various electrical loads. The difference in the thermal expansion coefficient between the core and the aluminum strands causes changes in tensile load distribution. As the temperature increases, the aluminum strands elongate more than the core, relaxing and transferring tensile load to the core. At a certain temperature, known as the knee point temperature (KPT), the tension on the aluminum strands is entirely transferred to the core, leaving no tensile load on the aluminum wires [119]. Due to the high tension experienced by the steel core within ACSS conductors, the reduction in tension force at elevated temperatures is minimal compared to ACSR conductors, resulting in lower overall sag. Moreover, as the KPT of ACSS is lower than that of ACSR due to the use of annealed aluminum strands, the sag response of an ACSS conductor is predominantly governed by the thermal characteristics of its steel core. The lower coefficient of thermal expansion of the steel core relative to the aluminum strands results in ACSS conductors exhibiting less sag at elevated temperatures than ACSR conductors. As a result, the sag–temperature gradient of an ACSS conductor is significantly less than that of an ACSR conductor of equivalent cross-sectional area [85,120].

### 5.10. Lightning Resistance

Lightning is a major cause of interruptions in overhead transmission lines in many countries [121]. Lightning strikes generate transient voltages that can exceed thermal limits, causing supply disruptions. Strikes exhibit peak currents ranging from 2 kA to 200 kA, with a 40 kA strike reaching temperatures of about 30,000 K and releasing 39.55 × 10^3^ J/Ω within microseconds [122]. When lightning strikes the upper conductor, it induces a high surge in current, potentially burning through the conductor’s outer layer. In shielding failure, lightning can bypass ground wires and directly strike phase conductors. The lightning current then splits into two, traveling in opposite directions along the transmission line, threatening system integrity [123]. To reduce lightning-induced trip-outs, several measures can be taken: incorporating ground wires, minimizing tower grounding impedance, increasing the Basic Lightning Insulation Level (BIL) of components, introducing underbuilt wires, and deploying line arresters [121].

### 5.11. Galloping

Ice formation on the conductor surface creates an imbalance, leading to aerodynamic instability. This imbalance lowers the natural frequency of the conductor, and when it aligns with a lower natural frequency for vertical vibrations, it triggers galloping [124]. Galloping is a wind-induced oscillation characterized by low frequency and high amplitude, affecting both individual and bundled conductors [125]. These oscillations, typically vertical, range from 0.1 to 1.0 times the sag between supports and vary in frequency depending on the line construction and oscillation mode. Galloping imposes elevated dynamic stresses on cables and support towers, causing mechanical damage such as loosening and expulsion of tower bolts, erosion of landing bolts, deformation of holes, and misalignment of tower pivots and connected components. Prolonged galloping induces fatigue in conductors and the steel framework of towers [125]. Methods to reduce power line vibrations include torsional devices, disrupting ice accretion and aerodynamic uniformity, mechanical dampers, de-spacing, rotating-clamp spacers, and optimized suspension and anchoring designs [124,125,126,127].

### 5.12. Aeolian Vibrations

Aeolian vibrations arise from the shedding of vortices when an object is exposed to laminar or turbulent flow [128]. In overhead transmission lines, wind-induced vortices cause vibrations within a 5 to 100 Hz frequency range, sometimes reaching amplitudes as large as the conductor’s diameter. These vibrations can cause significant damage and lead to conductor failure due to material fatigue, depending on wind conditions and the conductor’s geometrical and physical properties [98,129]. Accurate vibration assessments require a thorough understanding of wind data, wind power inputs, and the mechanical characteristics of conductors and damper components. To mitigate aeolian vibrations and extend conductor lifespan, various dampers, such as Stockbridge-type dampers, are used [130,131]. Analyzing the thermal and vibration characteristics of HTLS conductors is challenging due to significant differences between the core and outer layers. The core’s low thermal expansion and the distinct aluminum types and strand shapes of the outer layers contribute to these challenges. The installation, operation, and environmental conditions of the power line influence these complexities. HTLS conductors dissipate more power across the vibration frequency range, reducing the detrimental impacts of aeolian vibrations [132].

### 5.13. Fretting Fatigue

Fretting fatigue significantly threatens transmission line reliability, particularly at constrained points such as suspension clamps. Fretting fatigue is a complex process influenced by several factors, including normal contact load, amplitude of relative slip, coefficient of friction, surface conditions, contact materials, and environmental conditions. It results from the interaction of wear, corrosion, and fatigue phenomena driven by microslips at the contact surface and cyclic local stresses. Higher contact loads and increased slip amplitudes can exacerbate stress concentrations, while the friction coefficient affects stress distribution and heat generation. Surface roughness and material properties also play a critical role, with harsher environments accelerating material degradation. Together, these factors contribute to crack initiation and propagation, leading to material failure under cyclic loading conditions such as wind-induced vibrations [133]. Extensive research, including field studies, laboratory experiments, and simulations, has shown that fretting significantly contributes to fatigue-related failures in transmission line conductors [134,135,136,137,138,139,140,141,142,143,144,145]. Engineers and maintenance personnel must be aware of fretting issues and implement preventive measures to ensure the safe and reliable operation of these critical infrastructure components. Fatigue life predictions use S-N curves [139,143,146,147], and research indicates that higher conductor tensile strength increases fretting fatigue [139]. Additionally, even a small increase in mean stress significantly decreases fatigue life [143], highlighting the importance of considering tensile conditions in transmission line design and installation. While numerous studies have investigated fretting fatigue in ACSR and AAAC conductors, there is a notable lack of research on ACSS conductors. Interestingly, some studies have shown that annealed aluminum near the temper O condition exhibits an initial increase in fatigue life during the early stages of fatigue testing. This phenomenon has been attributed to an increase in dislocation density within the material. However, unlike strain-hardened aluminum wires, annealed aluminum wires typically exhibit lower overall fatigue resistance. This is likely because the annealing process reduces the strain-hardening effects known to be associated with improved fatigue strength. As a result, aluminum wires that have undergone strain hardening generally exhibit superior fatigue life performance compared to their annealed counterparts [144,148]. This suggests that the fatigue life of ACSS conductors may be less than that of ACSR conductors because ACSS conductors use annealed aluminum wires. Additionally, considering that fatigue failures typically occur in the aluminum strands rather than the steel core [85], this further contributes to the potential reduction in fatigue life for ACSS conductors.

Table 4 compares the key properties of ACSS with those of conventional ACSR conductors of the same outer diameter.

## 6. Environmental Effects and Sustainable Development

ACSS conductors offer advantages such as lower sag, higher ampacity, and a higher safety factor [151,152]. They are particularly suitable for reconductoring applications. Research shows that replacing HTLS conductors (including ACSS) with existing ACSR conductors significantly reduces environmental impacts compared to building new lines. Benefits include lower fossil fuel use, reduced greenhouse gas emissions, and less noise pollution. Additionally, less cement consumption contributes to reduced CO_2_ emissions. Establishing new lines requires maintaining a protection corridor, leading to deforestation [153]. HTLS conductors, including ACSS, reduce line losses due to the enhanced electrical conductivity of annealed aluminum, thus decreasing fuel consumption and greenhouse gas emissions. This supports clean energy delivery and aligns with emissions reduction initiatives [3]. A life cycle assessment (LCA) on electrical grids indicates that power losses dominate climate change impacts, followed by metal production for masts and conductors [154,155]. Reconductoring with ACSS can reduce these impacts due to their higher capacity. However, potential drawbacks include increased power losses and safety risks like fires from higher operating temperatures. The lack of a comprehensive LCA for ACSS conductors highlights the need for research on their environmental impacts throughout their life cycles.

## 7. Conclusions

This study extensively examined ACSS conductors to enhance their technical characteristics, focusing on efficiency in long-distance power transmission with minimal energy loss and resilience against environmental factors such as creep, corona, and sag. Manufacturing procedures for various ACSS conductors were detailed. The electrical, thermal, and mechanical behaviors and tests of ACSS conductors were introduced. The research identified existing deficiencies and standards, providing valuable insights for designers, researchers, and practitioners in power transmission projects. The primary objective was to improve the performance and dependability of ACSS conductors in power transmission applications.

## 8. Identified Challenges and Research Needs

Extensive research is needed on nano-coatings for developing superhydrophobic surfaces with stability at high temperatures, including the following areas:

1. Investigating the impact of various alloying elements on aluminum alloys’ mechanical and electrical properties at high temperatures is imperative;
2. Examining aging, fatigue, and corrosion of ACSS conductors with different configurations at high temperatures is essential;
3. Specialized investigation is required to understand how alloying elements affect steel alloys’ mechanical and electrical properties under high temperatures;
4. Developing specific standards for short-circuit tests on ACSS conductors is crucial, given the current IEC 60794 standard is tailored for OPGW conductors;
5. A comparative study assessing ACSS conductors versus other high-capacity conductors should include techno-economic analysis and losses;
6. Developing a dedicated standard test method to evaluate creep at high temperatures is essential;
7. Research on industrial-scale removal of impurities from aluminum melts is needed to enhance aluminum’s mechanical and electrical properties;
8. Selecting and incorporating suitable alloys into aluminum should be followed by a thorough examination of creep, fatigue, aging, and sag under high-temperature conditions, especially for novel ACSS conductors;
9. Comprehensive investigations are needed to understand the corrosion, creep, and fatigue behaviors of ACSS conductors with different core materials at high temperatures;
10. Evaluating ACSS conductors that have been in service for extended periods at high temperatures should include assessments of physical, mechanical, and electrical properties, as well as corrosion, fatigue, creep, and aging characteristics;
11. Further research is required on the mechanical, electrical, and thermal behavior of ACSS conductors and the development of specialized standards, given limited existing resources;
12. Research on the self-damping characteristics of ACSS conductors is needed due to the lack of comprehensive studies in this domain;
13. Addressing environmental impacts and promoting sustainable development of ACSS and other overhead conductors requires exhaustive life cycle investigations and comparative assessments with traditional ACSR and various HTLS conductors.

## Figures and Tables

**Table 1:** Chemical composition for steel wires used in the core of ACSS conductors [48,49,50,51,52].

Element	Composition, %		
	GA2/MA2	GA3/MA3	GA4/GA5/MA4/MA5
Carbon	0.5 to 0.88	0.5 to 0.88	0.5 to 1.00
Manganese	0.5 to 1.10	0.5 to 1.30	0.30 to 1.30
Phosphorus, max.	0.035	0.035	0.035
Sulfur, max.	0.045	0.045	0.045
Silicon	0.1 to 0.35	0.1 to 0.35	0.1 to 1.20

**Table 2:** Mechanical, physical, and thermal properties of Al and steel core used in ACSS conductors [48,49,50,51,52,54,55,56,57,58,59].

Wire Type	Standard	Density (kg/m^3^)	Electrical Conductivity (%IACS)	Coefficient of Linear Expansion (10^−6^ K^−1^)	Ultimate Tensile Strength (MPa)	Tensile Stress at 1% Elongation (MPa)	Elongation (%)
GA2	ASTM	7780	9.0	-	1240–1450	1070–1310	3.0–4.0
	BS EN	7780	-	11.5	1600–1700	1100–1170	3.0–4.0
GA3/HS	ASTM	7780	9.0	-	1520–1620	1340–1450	3.0–3.5
	BS EN	7780	-	11.5	1600–1700	1340–1450	2.0–2.5
GA4/EHS	ASTM	7780	9.0	-	1725–1825	1450–1550	3.0–3.5
	BS EN	7780	-	11.5	1600–1700	1340–1450	2.0–2.5
GA5/UHS	ASTM	7780	9.0	-	1825–1965	1480–1580	3.0–3.5
	BS EN	7780	-	11.5	1600–1700	1340–1450	2.0–2.5
MA2	ASTM	7780	9.0	-	1240–1450	1070–1310	3.0–4.0
MA3/HS	ASTM	7780	9.0	-	1520–1620	1340–1450	3.0–3.5
MA4/EHS	ASTM	7780	9.0	-	1725–1825	1450–1550	3.0–3.5
	BS EN	7780	-	11.5	1725–1825	1450–1550	3.0–3.5
MA5/UHS	ASTM	7780	9.0	-	1825–1965	1480–1580	3.0–3.5
	BS EN	7780	-	11.5	1825–1965	1480–1580	3.0–3.5
ACS/AW2	ASTM	6590	20.3	-	1103–1344	1000–1206	1.5
ACS/AW3	ASTM	6590	20.3	-	1340–1450	1170–1310	1.5
	BS EN	6590	20.3	13.0	1515–1620	1300–1390	1.5
Al 1350-O	ASTM	2705	61.8	-	60–95	-	-
	BS EN	2703	61.8	-	60–95	-	20.0
Al-Zr Alloy	ASTM	2700	60.0	23.0	155–165	-	2.0
	BS EN	2703	55.0–60.0	23.0	159–248	-	1.5–2.0

HS: high strength; EHS: extra-high strength; UHS: ultra-high strength; MA: zinc–5% aluminum mischmetal; ACS: aluminum-clad steel.

**Table 3:** Chemical composition for aluminum wires used in overhead conductors [38].

Element	Composition, %
Silicon, max.	0.10
Iron, max.	0.40
Copper, max.	0.05
Manganese, max.	0.01
Chromium, max.	0.01
Zinc, max.	0.05
Boron, max.	0.05
Gallium, max.	0.03
Vanadium plus titanium, total, max.	0.02
Other elements, each, max.	0.03
Other elements, total, max.	0.10
Aluminum, min.	99.50

**Table 4:** Comparison of key characteristics of ACSS with conventional ACSR conductors of the same outer diameter.

Parameter	ACSS to ACSR	Ref.
Maximum operating temperature	Much higher (up to 3 times higher)	[120,149]
Current-carrying capacity at maximum operating temperature	Much higher (up to 2 times higher)	[85,120,149]
Current-carrying capacity at same operating temperature	Higher (up to 25%)	[85,149]
Transmission losses at same operating temperature	Lower (up to 5%)	[85,149]
Creep strain	Lower	[85,149]
Temperature effect on sag	Much lower	[85,120]
Self-damping	Much higher (5 to 20 times)	[120,133,150]
Electrical conductivity at room temperature	Higher (up to 5%)	[85,149]
Tensile strength	Lower (up to 35 %)	[85,133,149]
Fatigue life	Lower probability	[144,148]
